# *TERT* promoter mutations and gene amplification: Promoting *TERT* expression in Merkel cell carcinoma

**DOI:** 10.18632/oncotarget.2491

**Published:** 2014-09-16

**Authors:** Hong Xie, Tiantian Liu, Na Wang, Viveca Björnhagen, Anders Höög, Catharina Larsson, Weng-Onn Lui, Dawei Xu

**Affiliations:** ^1^ Department of Oncology-Pathology, Cancer Center Karolinska; ^2^ Department of Pathology, Shandong University School of Medicine, Jinan, PR China; ^3^ Department of Reconstructive Plastic Surgery, Karolinska University Hospital Solna, Stockholm, Sweden; ^4^ Department of Medicine-Solna, Division of Hematology and Center for Molecular Medicine. Karolinska Institutet and Karolinska University Hospital Solna, Stockholm, Sweden

**Keywords:** Gene amplification, Merkel cell carcinoma, MCV, Promoter mutations, Telomerase, TERT

## Abstract

Telomerase activation through the induction of its catalytic component TERT is essential in carcinogenesis. The regulatory mechanism and clinical significance underlying cancer-specific *TERT* expression have been extensively investigated in various human malignancies, but little is known about these in Merkel cell carcinoma (MCC), an aggressive neuroendocrine skin tumor. Here we addressed these issues by determining *TERT* promoter mutations, gene amplification, mRNA expression and association with clinical variables in MCC. *TERT* mRNA was expressed in 6/6 MCC cell lines and 41 of 43 tumors derived from 35 MCC patients. Telomerase activity was detectable in all 6 cell lines and 11 tumors analyzed. *TERT* promoter mutations were identified in 1/6 cell lines and 4/35 (11.4%) MCC cases. The mutation exhibited UV signature and occurred in sun-exposed areas. Increased *TERT* gene copy numbers were observed in 1/6 cell lines and 11/14 (79%) tumors, and highly correlated with its mRNA expression (*r* = 0.7419, *P* = 0.0024). Shorter overall survival was significantly associated with higher *TERT* mRNA levels in MCC patients (*P* = 0.032). Collectively, *TERT* expression and telomerase activity is widespread in MCC, and may be attributable to *TERT* promoter mutations and gene amplification. Higher *TERT* expression predicts poor patient outcomes.

## INTRODUCTION

Merkel cell carcinoma (MCC), a neuroendocrine skin malignancy, is believed to originate from intraepidermal Merkel cells and predominantly occurs in the elderly population or immunocompromised individuals [1-4]. Although rare, the annual incidence of MCC has significantly increased in last decades [1-4]. Moreover, MCC is aggressive and has a mortality rate up to 33%, higher than that of any other skin tumors including malignant melanoma [3-5]. Immunosuppression, ultraviolet (UV) irradiation and infection of Merkel cell polyomavirus (MCV) have been proposed to contribute to the development of MCC [3-5], however, our current understanding of the disease pathogenesis remains limited. Clearly, profound insights into the MCC pathophysiology are required to develop novel therapeutic strategies for the management and outcome improvement of MCC patients.

Telomerase is a RNA-dependent DNA polymerase responsible for lengthening telomere and silent in most normal human cells with a limited life-span [6-10]. It is well established that activation of telomerase is an essential step in malignant transformation, and by stabilizing telomere length, telomerase confers transformed cells an infinite proliferation potential [6-10]. Indeed, numerous clinical observations have shown that telomerase activity is detectable in up to 90% of human malignancies [6-8]. Mechanistically, induction of telomerase reverse transcriptase (*TERT*) expression, the catalytic component of telomerase, is a key event activating telomerase [6, 9]. Thus, how cancer-specific *TERT* expression is achieved has long been an important issue in cancer research, but remains incompletely understood. The accumulated evidence has suggested that genetic factors play an important part in regulating TERT expression. We previously showed that *TERT* gene was frequently targeted for amplification in carcinogenesis, which contributed to telomerase activation in human malignancies [11, 12]. It has also been shown that certain single nucleotide polymorphisms increase cancer risk by up-regulating TERT expression and telomerase activity [13]. More recently, somatic *TERT* promoter mutations namely C228T and C250T have been identified as novel gain-of-function genetic events in up to 80% of malignant melanoma and other kinds of cancer [14-25]. All these findings, not only provide insights into telomerase activation in carcinogenesis, but also reveal clinical significance of telomerase/*TERT*-related assessments in cancer diagnosis and outcome prediction. Furthermore, targeting telomerase or telomere maintenance has been suggested as a novel anti-cancer strategy [26, 27].

Despite numerous existing data on telomerase/*TERT* in various types of human malignancies, little is known about these in MCC, and there has been so far only been one published report showing that telomerase activity was detected in 4/4 tumor biopsies from 4 MCC patients and in 3 of 4 cultured MCC cells derived from the above patients [28]. That result indicates widespread telomerase activation in MCC, however, further studies on large cohorts of patients are required to corroborate the finding. Moreover, it is currently unclear how telomerase is activated and how *TERT* expression is induced in MCC, and whether there exists a relationship between *TERT* expression and clinical-pathological features of MCC. In the present study, we address these issues by determining *TERT* expression, promoter mutation, gene amplification, telomerase activity and their clinical-pathological implications in MCC.

## RESULTS

Telomerase activation and association with clinical variables were analyzed together with *TERT* expression, promoter mutation, and gene amplification using a series of 43 MCCs from 35 patients (Table 1 and Supplementary Table S1) and 6 MCC cell lines.

**Table 1:** Summary of clinical features of 35 MCC patients

Characteristic (no. of informative)	No. of cases
No. of tumors	48
Gender (n = 35)	
Male	15
Female	20
Age at diagnosis (n = 35)	
Median (range) years	77 (20 - 100)
≤ 77 years	18
> 77 years	17
Type of lesion (n = 43)	
Primary	26
Local recurrence	7
Metastasis	10
Primary tumor size (n=31) (cm)	
Median (range) cm	2.5 (0.7 - 15)
≤ 2.5 cm	17
> 2.5 cm	14
Primary tumor location	
Head and neck	21
Arm	7
Other (thigh, gluteal region, groin, leg)	7
Survival (n = 34)	
<12 months	11
12-60 months	15
>60 months	8
Outcome (n = 34)	
Alive	9
Died of other causes	8
Dead of disease (DOD)	17
MCV status (n = 48)	
Positive	35
Negative	13
Type of materials (n = 48)	
FFPE	33
Frozen	15

FFPE = Formalin fixed paraffin embedded

### *TERT* mRNA expression and telomerase activity in MCC-derived cell lines and tumors from patients with MCC

**Figure 1:**
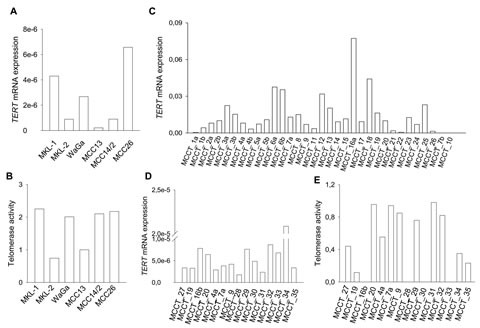
*TERT* mRNA expression and telomerase activity in MCC cell lines and tumors Relative *TERT* mRNA levels were expressed arbitrarily as the ratio of *TERT* and *18S* rRNA (Frozen samples) or ACTB (FFPE samples) CT values. The level of telomerase activity was expressed arbitrarily as folds of that in HEK-293 cells. (A) *TERT* mRNA expression and (B) telomerase activity in MCC cell lines. (C) *TERT* mRNA levels in 33 FFPE MCC tumors. (D) *TERT* mRNA levels in 15 frozen MCC tumors and (E) telomerase activity in those same tumors. Patients MCCT_16b, _28, _30 and _33 had no materials available for telomerase activity assessment.

A previous study reported detectable telomerase activity in tumor biopsies derived from 4 MCC patients [28], whereas *TERT* mRNA expression in MCC has not been investigated so far. Therefore, we first determined levels of *TERT* mRNA and/or telomerase activity in 6 MCC cell lines and 43 tumors [33 formalin-fixed, paraffin-embedded (FFPE) and 15 frozen samples; both FFPE and frozen tumor samples were available from 5 patients] obtained from 35 patients with MCC (Table 1 and Supplementary Table S1). Reverse transcription-quantitative PCR (RT-qPCR) analyses demonstrated the presence of *TERT* mRNA at different abundances in all 6 examined MCC cell lines, and 15/15 frozen and 31/33 FFPE tumor specimens (Fig. 1A, C and D, Table 2 and Supplementary Table S2 and S3). Thus, a total of 41 tumors derived from 34 MCC patients (34/35, 97%) expressed *TERT* mRNA. Consistent with *TERT* expression, telomerase activity was detected in all 6 cell lines and 11 MCC frozen tumors analyzed (Fig. 1B and E, Table 2 and Supplementary Table S2). As expected, the cell lines exhibited higher telomerase activity than did MCC tumors (Fig. 1B and E, and Supplementary Table S2). In the activity assay, telomerase-positive HEK-293 cell-derived extract and its heat-treated counterpart were analyzed in parallel as positive and negative controls, respectively. In addition, adjacent normal skin tissues derived from a MCC patient was also included and demonstrated their absence of telomerase activity (data not shown).

**Table 2:** Summary of TERT alterations and telomerase activation in MCC tumors and cell lines

Parameter analyzed	MCC	MCC
(number of informative MCC samples)	tumors	cell lines
Total number of patients	35	6
Total number of samples	48	6
		
*TERT* mRNA expression (n = 48)		
Positive	46 / 48	6 / 6
Negative	2	0
		
Telomerase activity (n = 11)		
Positive	11 / 11	6 / 6
Negative	0	0
		
*TERT* promoter sequence (n = 48)		
Mutated	5 / 48	1 / 6
Wild-type	43 / 48	5 / 6
		
*TERT* copy numbers (n = 14 )		
Gain	11 / 14	1 / 6
Normal	2 / 14	5 / 6
Loss	1 / 14	0

### *TERT* promoter mutations in MCC

**Figure 2:**
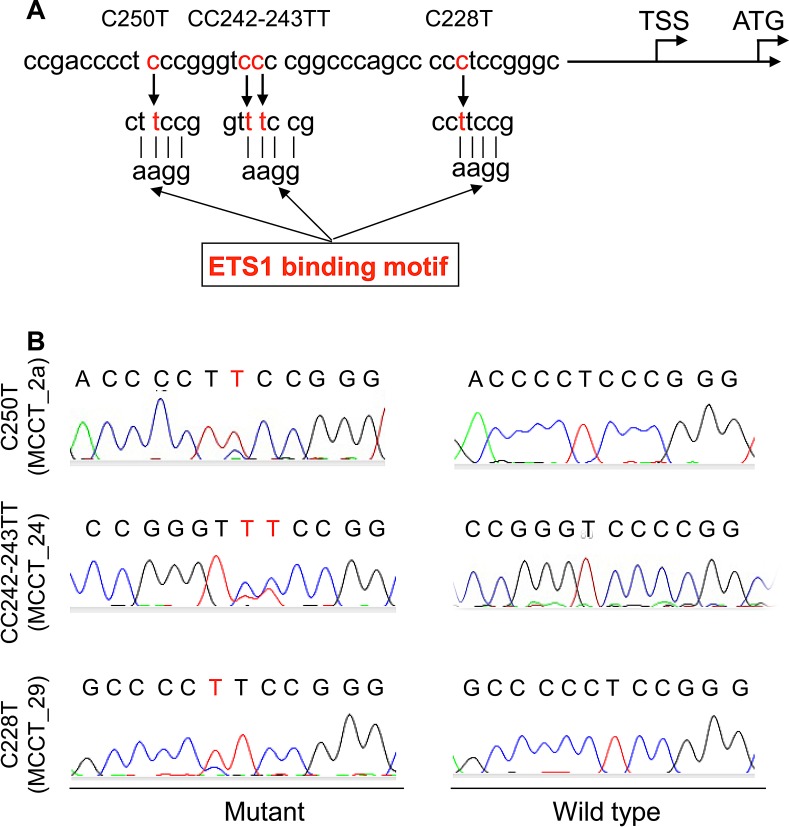
*TERT* promoter mutations identified in tumors derived from patients with MCC (A) Schematic illustration showing the location of C228T, CC242-243TT and C250T (in red) in the *TERT* core promoter. Each of these mutations generates an extra ETS binding motif on the *TERT* proximal promoter. TSS and ATG: Transcription and translation start sites, respectively (Accession number: AF128893.1). (B) Sequencing chromatographs of the *TERT* promoter locus in tumor DNA- (left panel) and peripheral blood DNA (right panel) from patients with MCC obtained by Sanger sequencing. Chromatograms shown are C to T transitions at 250 (Top) and 228 (Bottom), and CC to TT alterations at 242-243 (middle) in the *TERT* promoter.

The above result reveals a widespread *TERT* expression and telomerase activation in MCC. Given the recent finding that the UV-related *TERT* promoter mutation, up-regulating *TERT* gene transcription, widely occurs in malignant melanoma and other skin cancers [16], we sought to ask whether this was also the case in MCC. *TERT* promoter sequencing was performed on 6 MCC cell lines and 43 tumor specimens from 35 patients with MCC. The mutation was found in 1/6 MCC cell lines and 5 tumors (MCCT_2a, MCCT_2b, MCCT_17; MCCT_24 and MCCT_29) derived from 4 patients (4/35, 11.4%) (Fig. 2, Table 2 and Supplementary Table S1), revealing the involvement of this genetic event in the MCC pathogenesis, although the frequency was lower than that in malignant melanoma. The mutant cell line harbored a C250T. One patient (MCCT_2) had the C250T mutation in the tumors from both diagnosis and recurrence. The three remaining mutation-carrying tumors exhibited C250T, C228T and CC242-243TT, respectively. C250T was the major type of *TERT* promoter mutations identified in MCC, which is different from most reported malignancies. Of note, for 3 of 4 mutation-carrying tumors, the primary tumor was located in the face and the remaining one was at the temple. Both face and temple are UV-exposed areas in the body and there was a significant difference in *TERT* promoter mutations between tumors at these sun-exposed and other areas (*P* = 0.035, Fisher’s exact test).

### The *TERT* gene amplification in MCC tumors

The *TERT* gene is localized in chromosomal region 5p15.33 whereas gains of this region are prevalent in MCC [12, 29, 30]. Because the *TERT* amplification was previously found in many kinds of cancer [11, 12], we examined *TERT* gene copy numbers in 6 MCC cell lines and 14 patient-derived frozen tumors using qPCR. Patients’ peripheral blood leukocytes and *TERT*-amplified HeLa cell line [12] were used as normal (2 copies/cell) and positive (5 copies/cell) controls, respectively. One of 6 cell lines was found to harbor 5 *TERT* copies/cell and the remaining 5 lines had normal numbers (Table 2 and Supplementary Table S2). Eleven of 14 examined MCC tumors exhibited increased *TERT* copies ranging from 3 to 12/cell, whereas 1 of them had only one *TERT* copy each cell, indicating its deletion (Supplementary Table S2). Normal *TERT* copy numbers were observed in 2 tumors (Supplementary Table S2). The presence of *TERT* amplification was not related to anatomic sites of MCC, which was different from the *TERT* promoter mutation.

### Correlation between *TERT* mRNA levels and *TERT* gene amplification in MCC tumors

**Figure 3:**
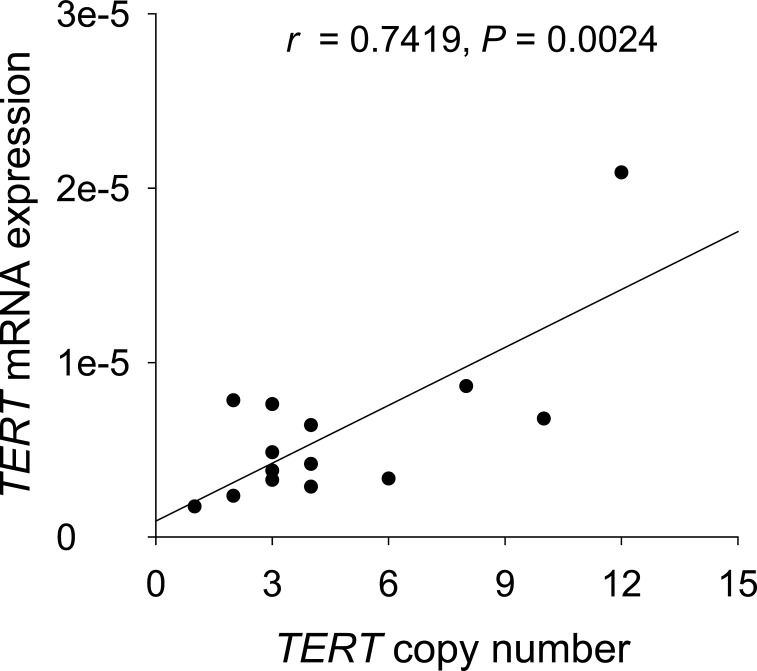
Positive correlation between *TERT* copy numbers and *TERT* mRNA abundance in MCC tumors The scatter plot shows results for *TERT* mRNA and gene copy number analyses by RT-qPCR in 14 fresh frozen MCC samples. *TERT* mRNA expression was significantly correlated with *TERT* copy numbers (*r* = 0.7419, *P* = 0.0024).

To see whether the *TERT* amplification plays a functional role in *TERT* mRNA expression, we made a correlation analysis between the *TERT* copy number and its mRNA level in 14 tumors. As shown in Fig. 3 and Supplementary Table S2, higher *TERT* mRNA expression was significantly correlated with increased *TERT* copies in primary MCC tumors (*r* = 0.7419, *P* = 0.0024), suggesting a positive effect of the *TERT* amplification on gene transcription.

### Relationship of MCV with the *TERT* promoter mutation, gene amplification or mRNA expression

MCV is implicated in the pathogenesis of MCC [31], and we were thus interested in the relationship between MCV and the *TERT* promoter mutation and *TERT* expression. In our cohort, 3/6 (50%) MCC cell lines and 25/35 (71%) MCC tumors were MCV-positive (Table 1 and Supplementary Table S1 and S2). The one cell line with *TERT* promoter mutation was negative for MCV. Three of the 4 mutation-carrying MCC tumors were MCV-negative and one was MCV-positive (Supplementary Table S2 and S3). These results suggest that the *TERT* promoter mutation tends to occur in MCV-negative MCCs (mutations in MCV+ vs - cases: *P* = 0.0613, Fisher’s exact test). When both cell lines and patients were analyzed together, there was a significant difference in the mutation between tumors with and without MCV (*P* = 0.011, Fisher’s exact test). There was no difference in *TERT* mRNA expression either between MCV+ and – cell lines or patients’ tumors. We were unable to perform a correlation analysis on MCV status and *TERT* gene copies due to too few (only one) MCV-negative tumors.

### Clinical relevance of the *TERT* promoter mutation, gene amplification and mRNA expression in MCC

**Figure 4:**
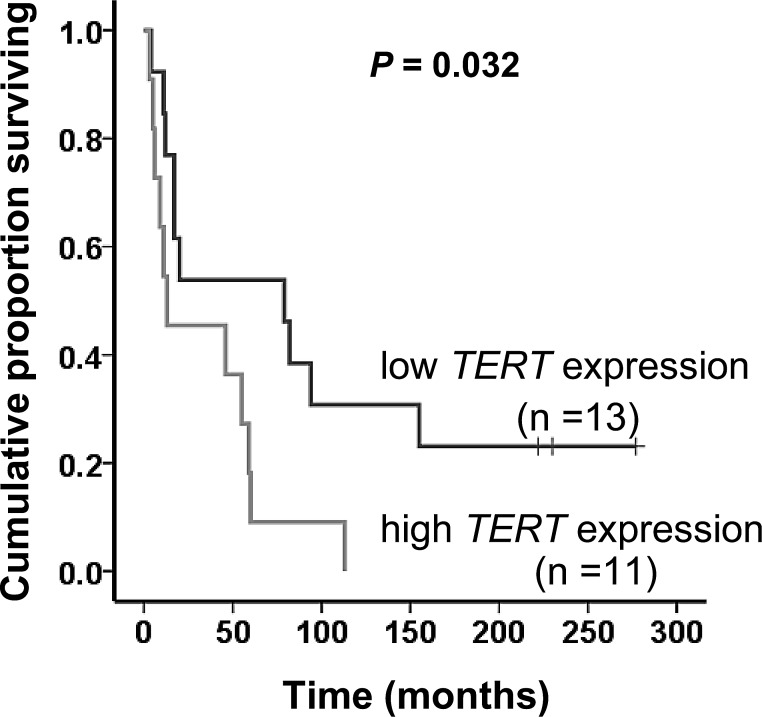
Significant correlation between the *TERT* mRNA level and overall survival in MCC patients The level of *TERT* mRNA expression in 24 evaluable MCC patients was grouped into low and high categories (lower and higher than median values, respectively) and the relationship of patients’ overall survival with *TERT* expression was assessed using Kaplan-Meier plots and significance was determined using log-rank (Mantel –Cox) test.

Age at diagnosis, gender, tumor size, metastasis and recurrence were not associated with the *TERT* promoter mutation and gene amplification (Supplementary Table S4). However, higher *TERT* mRNA expression was more frequently observed in male patients (*P* = 0.047). In 24 evaluable MCC patients, lower levels of *TERT* mRNA expression in tumors were significantly associated with longer overall survival time [*P* = 0.032, Log-rank (Mantel-Cox) test; Fig. 4].

## DISCUSSION

In the present study, we performed a comprehensive analysis on the genetic regulation of *TERT* expression and its clinical-pathological association in MCC. Our results demonstrate that (i) *TERT* expression and telomerase activity is highly prevalent in MCC; (ii) *TERT* promoter mutations are identified in > 10% of MCC, and most frequently occur at sun-exposed areas and MCV-negative tumors; (iii) The *TERT* gene amplification is widespread and positively correlated with *TERT* expression levels in MCC; and (iv) Higher levels of *TERT* mRNA expression are significantly associated with shorter survival time in MCC patients. These findings provide important insights into the regulatory mechanism underlying telomerase activation in MCC and may be implicated in future MCC management.

The recurrent *TERT* promoter mutations create *de novo* ETS binding motifs, thereby facilitating the *TERT* transcription and activating telomerase, and they have been identified in various types of human malignancies [19]. However, the mutation frequency varies substantially from cancer to cancer. They are widespread in melanoma, cutaneous base and squamous cell carcinoma, bladder and renal pelvic cancer, hepatocellular carcinoma, glioblastoma and certain thyroid cancer, while rare or absent in prostate, lung, breast and digestive track cancer, and hematological malignancies [14-16, 18-21, 23]. In the present study, we identified the *TERT* promoter mutation in 1/6 MCC cell lines (16.7%) and 4/35 patients with MCC (11.4%), which suggests that it is not a rare genetic event in MCC. All the identified mutations, including C250T, C228T and CC242-243TT, lead to gain-of-function via the creation of an extra ETS binding motif [19]. However, the mutation profile observed in MCC is very unique. The C250T mutation was predominant in MCC (60%), which is in sharp contrast to almost all the reported human malignancies where C228T is much more prevalent [15, 16, 19, 25]. In addition, the CC242-243TT mutation, observed in 1/4 of MCC tumors, is rarely seen in other types of cancer (<4% and 0.5% in melanoma and bladder cancer, respectively). It will be interesting to probe what causes such a difference in the *TERT* promoter mutation pattern between MCC and other human malignancies. It should be pointed out, however, that we are unable to exclude potential sampling error and thus the result obtained from this cohort of 35 patients is unlikely conclusive. Further studies recruiting more MCC patients are required to confirm the present finding.

Up to 80% of malignant melanoma harbor *TERT* promoter mutations and UV irradiation was proposed to result in these mutagenic lesions [15, 16, 25]. UV irradiation is also closely associated with the pathogenesis of MCC, and our findings do support a potential link between the *TERT* promoter mutation and UV lesions. First, C to T and especially CC to TT alterations at the *TERT* promoter, observed in MCC, represent the hallmark of UV-induced DNA mutagenesis. Second, the *TERT* promoter mutation occurs exclusively in MCC tumors localized at face or temple, sun-exposed areas. These features were similarly observed in malignant melanoma and other skin cancers [15, 16, 25].

Compared to the *TERT* promoter mutation, the *TERT* amplification is more prevalent in MCC. We found that 11 of 14 examined MCC tumors (79%) harbored the increased *TERT* copy number. This result is consistent with previous cytogenetic and CGH data showing the frequent regional or whole arm gains of 5p where the *TERT* locus is localized [4, 29, 30]. Unlike the promoter mutation, the *TERT* amplification occurs in both sun-exposed and other areas, reflecting its irrelevance with UV irradiation [4, 29, 30]. Notably, there exists a highly positive correlation between the *TERT* gene copy number and its mRNA level, which indicates a functional impact of the *TERT* amplification on the *TERT* transcription in MCC. Taken together, the *TERT* gene amplification plays a significant role in telomerase activation during the development of MCC.

A number of oncogenic viruses target the *TERT* gene by de-repressing its transcription for telomerase activation [32, 33]. Because MCV is frequently detected in MCC and implicated in the disease pathogenesis, we are interested in a potential association between the virus infection and *TERT* promoter mutation or gene amplification. Intriguingly, the *TERT* promoter mutation tends to occur in MCV-negative tumors. This is similar to that observed in hepatocellular carcinomas where the *TERT* promoter mutation is significantly more frequent in patients without hepatitis virus B infection [18]. Owing to the limited number of patients, the relationship between the virus status and *TERT* amplification remains to be defined. It is currently unclear whether MCV, like other viruses, regulates *TERT* expression at the transcriptional level. SV40 was shown to activate telomerase in human mesothelial cells via its small T (sT) [34], and given a similar sT encoded by MCV, it could also be the case in the virus-positive MCC. Further experimental studies are required to answer this important question.

Clinical observations suggest that *TERT* promoter mutations may predict outcomes and associate with aggressive diseases in a number of cancer types [19]. In the present study, we identified 4 MCC patients with the *TERT* promoter mutation in their tumors, and the number is not sufficient to determine its prognostic or clinical power. Further investigations on a larger cohort of patients are required to address this issue. Nevertheless, a significant association between higher *TERT* expression and shorter patients’ survival observed in the present study indicates that *TERT* may serve as prognostic marker and be a therapeutic target for MCC.

In summary, the study presented here reveals a widespread *TERT* expression and telomerase activation in MCC. The *TERT* gene amplification and promoter mutation may significantly contribute to the de-repression of *TERT* transcription, whereas higher levels of *TERT* expression consequently contribute to poor patients’ outcomes. Moreover, given multi-biological activities of *TERT*/telomerase in cancer development and progression, [35] and telomerase-based cancer therapy as a novel anti-cancer strategy [26], it is worth of determining whether the combination of conventional therapeutic approaches with telomerase inhibitors is capable of improving treatment efficacy and survival in MCC patients.

## MATERIALS AND METHODS

### Patient specimens and MCC cell lines

A total of 48 tumor specimens from 35 patients with MCC were collected and most of them (33/48) had been described in a recent report [36] (Table 1 and Supplementary Table S1). There were 20 female and 15 male patients, with a median age at diagnosis of 77 years (range 20 to 100). Out of the 48 tumors, 33 were obtained as FFPE samples and 15 were fresh-frozen. For 7 patients, matched pairs of primary and recurrent tumors were available. All these MCC specimens exhibited high tumor content with >80% tumor cells. The MCC diagnosis was based on histopathological and immunohistochemical examinations. All the patients were followed-up until March 2014 or until death. Detailed clinical and histopathological information is given in Supplementary Table S1. The study was approved by the local Ethics Committee at Karolinska Institutet.

Six MCC cell lines were studied. Three of them were MCV-positive and the remaining 3 were MCV-negative. The MCV-positive cell lines WaGa, MKL-1 and MKL-2 were kindly provided by Drs. Jürgen C. Becker (Medical University of Graz, Graz, Austria), Nancy L. Krett (Northwestern University, Chicago, IL) and Roland Houben (University Hospital Würzburg, Würzburg, Germany), respectively. The MCV-negative MCC cell lines MCC13, MCC14/2 and MCC26 were purchased from CellBank Australia (Westmead, Australia). Cells were grown in RPMI 1640 medium containing 10% (WaGa, MKL-1 and MKL-2) or 15% (MCC13, MCC14/2 and MCC26) fetal bovine serum, and 2 ml L-glutamine under 37 ºC / 95% air / 5% CO_2_.

### DNA extraction, *TERT* promoter sequencing and *TERT* gene copy number determination

Genomic DNA was extracted from FFPE tissues and frozen tumors or cell lines using QIAmp DNA FFPE Tissue kit and Qiagen DNeasy Blood and Tissue kit (Qiagen, Hildane, Germany), respectively. The two common mutations C228T and C250T in the *TERT* proximal promoter correspond to positions 124 and 146 bp upstream of the ATG site, while the CC242-243TT mutation was at 137 and 138 bp. The target region covering these mutations (from 214 bp to 22 bp upstream of the ATG site) were amplified using conventional PCR followed by Sanger sequencing as described [17]. The PCR was performed with the following primer pairs: 5′-CACCCGTCCTGCCCCTTCACCTT-3′ and 5′- GGCTTCCCACGTGCGCAGCAGGA-3′. The C228T, CC242-243TT and C250T mutations were verified by sequencing from both directions.

*TERT* gene copy numbers were quantified using qPCR with the primer pair described above. *β-globin* gene was PCR-amplified in parallel as a reference for normalization, using the primers 5′-TGTGCTGGCCCATCACTTTG-3′ (forward) and 5′-ACCAGCCA-CCACTTTCTGATAGG-3′ (reverse). *TERT* copy numbers in peripheral blood DNA (2 copies/cells) from MCC patients or normal individuals and HeLa cells (5 copies/cell), well-defined in our previous study,[12] were used as normal and positive controls, respectively. Two independent assays were performed and the *TERT* copy number was determined by mean Ct values from two independent assays.

### RNA extraction and RT-qPCR for *TERT* mRNA expression

Total cellular RNA was extracted from frozen and FFPE specimens using mirVana miRNA isolation kit (Life Technology) and modified TriZol method, respectively [36]. qPCR was carried out using an ABI 7900HT Real time PCR System (Applied Biosystems) and Taq Man Gene Expression Assays (Applied Biosystems) for *TERT* (Hs00972656_m1), *ACTB* (Hs01060665_g1) and *18S* rRNA (Hs99999901_s1). Expression levels of *TERT* mRNA were calculated from threshold cycle values and normalized to *18S* and *ACTB* values for frozen and FFPE tissues, respectively.

### Telomerase activity assessment

Telomerase activity was determined using a TeloTAGGG Telomerase PCR ELISA kit (Roche Diagnostics GmbH, Mannheim, Germany). One microgram protein was used in each assay. Reaction mixtures with human embryonic kidney (HEK)-293 cell protein extracts and their heat-inactivated counterparts were used as positive and negative controls, respectively. Telomerase activity was calculated from the absorbance at optical density (OD) OD450-OD690 and expressed as the folds of that in HEK-293 cells.

### MCV detection

MCV detection was performed using PCR and/or immunohistochemistry [31, 36-38]. The detection of MCV status in all FFPE samples has been previously published [36], while all 15 frozen tumors were characterized in this study.

### Statistical analyses

The Pearson correlation analysis was used to determine correlation between the *TERT* gene copy number and mRNA level. High and low *TERT* expression groups were defined by the median expression levels of the tumors analyzed. Differences in the *TERT* promoter mutation frequency and *TERT* mRNA expression between tumors with gender, clinical stage, tumor size, metastasis and MCV status were determined using Fisher's exact test. Overall survival was illustrated by Kaplan-Meier plots, and significance was calculated by log-rank (Mantel –Cox) test. All the tests were two-tailed and computed using Statistica 7.0 software (StatSoft, Tulsa, OK). *P* values of <0.05 were considered as statistically significant.

### Disclose any potential conflicts of interest

The authors disclose no conflicts of interest.

## Supplementary Material


