# Supplementary material for: TERT promoter mutations and gene amplification: Promoting TERT expression in Merkel cell carcinoma
Source: Oncotarget. 2014 Sep 16;5(20):10048–57. doi: 10.18632/oncotarget.2491 (PMC4259404; doi:10.18632/oncotarget.2491)
Supplement: Supplementary file 1 [file oncotarget-05-10048-s001.pdf]

TERT promoter mutations and gene amplification: Promoting *TERT* expression in Merkel cell carcinoma

Supplementary Material

Supplementary Table S1. Clinical details, follow-up, MCV and *TERT* promoter statuses of MCC cases in the study.

Case no.[1]	*TERT* promoter sequence	Sex	Age at diagnosis (years)	Sample analyzed: tumor	Sample analyzed: material	MCV status[2]	Primary tumor: Size (cm)	Primary tumor: Anatomic site	Local Rec	Met	Follow-up: Time (months)	Follow-up: Outcome
MCCT_1a	wt	F	91	P	FFPE	-	2	face	no	yes	79	Dead
MCCT_1b	wt			Met	FFPE	-						
MCCT_2a	C250T	F	91	P	FFPE	-	1.5	face	yes	yes	20	Dead-DOD
MCCT_2b	C250T			L Rec	FFPE	-						
MCCT_3a	wt	F	83	P	FFPE	+	1.5	face	no	yes	6	Dead-DOD
MCCT_3b	wt			Met	FFPE	+						
MCCT_4a	wt	M	69	P	FFPE & Frz	+	3.5	elbow	no	yes	17	Dead-DOD
MCCT_4b	wt			Met	FFPE	+						
MCCT_5a	wt	F	84	P	FFPE	+	1	face	yes	yes	11	Dead-DOD
MCCT_5b	wt			L Rec	FFPE	+						
MCCT_6a	wt	M	74	P	FFPE	+	4.5	elbow	yes	no	113	Dead
MCCT_6b	wt			L Rec	FFPE	+						
MCCT_7a	wt	F	87	P	FFPE & Frz	+	2.5	face	yes	yes	13	Dead-DOD
MCCT_7b	wt			Met	FFPE	+						
MCCT_8	wt	F	85	P	FFPE	+	3.5	forearm	n.a.	n.a.	n.a.	n.a.
MCCT_9	wt	M	67	P	FFPE & Frz	+	2.3	face	no	no	17	Dead
MCCT_10	wt	F	70	P	FFPE	+	n.a.	thigh	no	no	1	Dead-DOD
MCCT_11	wt	F	91	P	FFPE	+	15	gluteal region	no	no	4	Dead-DOD
MCCT_12	wt	M	77	P	FFPE	-	1	face	yes	yes	11	Dead-DOD
MCCT_13	wt	M	84	P	FFPE	-	2.5	scalp	no	yes	3	Dead-DOD
MCCT_14	wt	F	85	P	FFPE	+	3	face	no	no	155	Dead
MCCT_15	wt	F	77	P	FFPE	+	1.8	forearm	no	no	55	Dead
MCCT_16a	wt	M	62	P	FFPE	-	3	groin	yes	yes	5	Dead-DOD
MCCT_16b	wt			Met	frz	-						
MCCT_17	C250T	M	77	P	FFPE	-	2	face	no	yes	12	Dead-DOD
MCCT_18	wt	M	71	P	FFPE	+	3	gluteal region	no	no	60	Dead
MCCT_19	wt	M	76	L Rec	FFPE & Frz	+	6	gluteal regior	yes	no	9	Dead-DOD
MCCT_20	wt	F	46	L Rec	FFPE & Frz	+	1	arm	yes	yes	230	Alive
MCCT_21	wt	F	20	L Rec	FFPE	-	10	scalp	yes	no	277	Alive
MCCT_22	wt	F	63	Met	FFPE	+	5	gluteal region	no	yes	222	Alive
MCCT_23	wt	M	81	Met	FFPE	-	1	scalp	no	yes	46	Dead-DOD
MCCT_24	CC241-242TT	F	72	Met	FFPE	-	0.7	face	no	yes	82	Dead-DOD
MCCT_25	wt	M	84	Met	FFPE	+	0.8	earlobe	yes	yes	59	Dead
MCCT_26	wt	F	85	Met	FFPE	-	n.a.	face	no	yes	94	Dead
MCCT_27	wt	F	89	P	Frz	+	4	face (chin)	no	yes	4	Dead-DOD
MCCT_28	wt	M	94	P	Frz	+	5	scalp	yes	no	17	Dead-DOD
MCCT_29	C228T	M	71	P	Frz	+	2.2	temple	no	yes	36	Alive
MCCT_30	wt	F	75	P	Frz	+	1	arm	no	no	35	Alive
MCCT_31	wt	M	83	P	Frz	+	n.a.	arm (wrist)	no	no	31	Alive
MCCT_32	wt	F	87	P	Frz	+	3.1	temple	yes	yes	5	Dead-DOD
MCCT_33	wt	M	75	L Rec	Frz	+	0.9	face (cheek)	yes	yes	18	Alive
MCCT_34	wt	F	73	P	Frz	+	3.5	leg	no	yes	12	Alive
MCCT_35	wt	F	100	P	Frz	+	n.a.	face (cheek)	yes	yes	4	Alive

[1] a and b = primary and recurrent tumor, respectively, of the same patient

2 MCV status was determined by PCR of tumor genomic DNA combined with IHC for MCV large T-antigen expression

P = Primary tumor; Met = Metastasis; L Rec = Local Recurrence; Frz = Frozen

F = female; M = male; - = negative; + = positive; DOD = died of disease; n.a. = not available

Supplementary Table S2. Details of telomerase activity, *TERT* mRNA expression, promoter mutation, gene copy number and MCV status in frozen MCC tissues and cell lines.

Case no.	telomerase activity[1]	*TERT* mRNA expression[2]	*TERT* copy number	*TERT* promoter	MCV status
MCC tumors					
MCCT_27	0.44	3,346053E-06	6	wt	+
MCCT_19	0.12	3,26972E-06	3	wt	+
MCCT_16b	n.a.	7,83803E-06	2	wt	-
MCCT_20	0.96	6,41E-06	4	wt	+
MCCT_4a	0.55	2,8825E-06	4	wt	+
MCCT_7a	0.94	3,8134E-06	3	wt	+
MCCT_9	0.85	4,19244E-06	4	wt	+
MCCT_28	n.a.	1,75341E-06	1	wt	+
MCCT_29	0.75	7,61933E-06	3	C228T	+
MCCT_30	n.a.	4,86471E-06	3	wt	+
MCCT_31	0.98	2,35537E-06	2	wt	+
MCCT_32	0.82	8,65326E-06	8	wt	+
MCCT_33	n.a.	6,79139E-06	10	wt	+
MCCT_34	0.35	2,08505E-05	12	wt	+
MCCT_35	0.23	3,35682E-06	n.a.	wt	+
Established Cell lines					
MCC13	0.99	2,1141E-07	2	wt	-
MCC14/2	2.11	8,98708E-07	2	wt	-
MCC26	2.17	6,5694E-06	5	C250T	-
MKL-1	2.25	4,29874E-06	2	wt	+
MKL-2	0.74	8,90906E-07	2	wt	+
WaGa	2.01	2,67291E-06	2	wt	+

[1] Telomerase activity was expressed as folds of that in HEK-293 cells

[2] *TERT* mRNA expression was determined by qPCR, normalized to *18S rRNA* and reported as $2^{-\Delta C_T}$.

Supplementary Table S3. Details of *TERT* mRNA expression, promoter mutation and MCV status in FFPE MCC tumors.

Case no.	*TERT* mRNA expression[1]	*TERT* promoter	MCV status
MCCT_1a	0.0005	wt	-
MCCT_1b	0.0043	wt	-
MCCT_2a	0.0081	C250T	-
MCCT_2b	0.0101	C250T	-
MCCT_3a	0.0224	wt	+
MCCT_3b	0.0153	wt	+
MCCT_4a	0.0081	wt	+
MCCT_4b	0.0032	wt	+
MCCT_5a	0.0073	wt	+
MCCT_5b	0.0108	wt	+
MCCT_6a	0.0376	wt	+
MCCT_6b	0.0353	wt	+
MCCT_7a	0.0129	wt	+
MCCT_7b	N.D.	wt	+
MCCT_8	0.0151	wt	+
MCCT_9	0.0070	wt	+
MCCT_10	N.D.	wt	+
MCCT_11	0.0035	wt	+
MCCT_12	0.0318	wt	-
MCCT_13	0.0203	wt	-
MCCT_14	0.0091	wt	+
MCCT_15	0.0115	wt	+
MCCT_16a	0.0773	wt	-
MCCT_17	0.0092	C250T	-
MCCT_18	0.0441	wt	+
MCCT_19	0.0163	wt	+
MCCT_20	0.0010	wt	+
MCCT_21	0.0018	wt	-
MCCT_22	0.0006	wt	+
MCCT_23	0.0126	wt	-
MCCT_24	0.0070	CC241-242TT	-
MCCT_25	0.02303	wt	+
MCCT_26	0.0014	wt	-

[1] *TERT* mRNA expression was determined by RT-qPCR, normalized to *ACTB* and reported as $2^{-\Delta C_T}$.

N.D. = not detectable

Supplementary Table S4. Association of TERT expression levels (in FFPE samples), TERT promoter mutation and TERT copy number with clinical features in MCC patients.

	TERT mRNA expression (only FFPE)			*TERT* promoter mutation			*TERT* gene amplification		
**Clinical parameter**	**Low**	**High**	***P*-value***	**Yes**	**No**	***P*-value***	**Yes**	**No**	***P*-value***
Number of MCC patients	13	12		4	31		11	3	
Number of MCC samples	16	15		5	38		11	3	
Gender			**0.047**			1.000			0.209
Male	3	8		2	13		5	3	
Female	10	4		2	18		6	0	
Age at diagnosis			1.000			0.603			0.506
Median (range) years: 77 (20 - 100)									
≤ 77 years	7	6		3	15		8	1	
> 77 years	6	6		1	16		3	2	
Primary tumor size			1.000			0.107			0.462
Median (range) cm: 2.5 (0.7 - 15)									
⩽ 2.5 cm	7	7		4	13		6	0	
> 2.5 cm	5	5		0	14		5	2	
Recurrence/ metastasis			1.000			0.551			1.000
Yes	10	9		4	22		9	2	
No	3	2		0	8		2	1	
MCV status			0.273			**0.024**			0.214
Positive	8	11		1	29		11	2	
Negative	8	4		4	9		0	1	

* P-values were determined by Fisher's exact test.
